# Prevention of Infection in Pregnancy

**DOI:** 10.1155/S1064744997000252

**Published:** 1997

**Authors:** William J. Ledger, Daniel Skupski

**Affiliations:** 1 Department of Obstetrics and Gynecology Cornell University Medical College New York NY USA; 2 Department of Obstetrics and Gynecology New York Hospital-Cornell Medical Center 525 East 68th Street, J-130 New York NY 10021 USA

## Abstract

We believe the prevention of infection-related adverse pregnancy outcome is the most important focus for obstetricians today. An emphasis upon immunization of susceptible women, prevention of transmissible disease by modification of patient behavior, and identification and treatment of silent infections should become standards of practice. This will require educational initiatives for physicians and their patients as well as continued clinical trials to determine costs and effectiveness.


Infectious Diseases in Obstetrics and Gynecology 5:165–175 (1997)
© 1997 Wiley-Liss, Inc.

### Prevention of Infection in Pregnancy

**William J. Ledger* and Daniel Skupski**
*Department of Obstetrics and Gynecology, Cornell University Medical College, New York, NY*

### Abstract

**We believe the prevention of infection-related adverse pregnancy outcome is the most important focus for obstetricians today. An emphasis upon immunization of susceptible women, prevention of transmissible disease by modification of patient behavior, and identification and treatment of silent infections should become standards of practice. This will require educational initiatives for physicians and their patients as well as continued clinical trials to determine costs and effectiveness. Infect. Dis. Obstet. Gynecol. 5:165–175, 1997.** © 1997 Wiley-Liss, Inc.

Key words
infection-related adverse pregnancy outcome; prevention; silent infections

This is an uncomfortable topic for obstetricians. It requires a change in practice priorities. As a specialty, we have focused upon operative skills plus the recognition and treatment of disease. Moving our priorities forward to prevention will be difficult because it represents an abrupt change in care. What are our standards today? In middle class America, the newly sexually active teenager switches her physician provider from the pediatrician to the obstetrician. Currently, the screening for this young woman is a Papanicolaou (Pap) smear and prevention is limited to a prescription for an oral contraceptive. This protects against an unwanted pregnancy, but offers no protection against sexually transmitted diseases. For urban poor women, no preventive care is available. The focus is on pathology. We treat urban poor teenagers with an unwanted pregnancy or symptomatic pelvic inflammatory disease. This is a narrow and unacceptable scope of practice.

An outline for infection-related adverse pregnancy outcomes has been the mnemonic TORCH, in which the significant pathogens for the fetus have been identified. We would like to expand this to STORCH[5] for the purposes of this discussion of prevention (see Table 1). The focus will be directed toward three strategies of prevention: vaccine administration, preventive counseling to achieve maternal behavior modification, and treatment of silent infections. All of these strategies require the use of screening laboratory studies to determine the risks in asymptomatic patients. This requires, on the part of the obstetrician, knowledge and understanding of laboratory studies, many of which are new.

### VACCINES

An awareness of immunization standards is important for the obstetrician. In the United States, we are the primary care physician for most women during their childbearing years. We need to know the needs of our patients and the status of vaccines, i.e., are they live virus preparations or not. For the pregnant woman, live virus vaccines should be avoided.

### Tetanus-Diphtheria

This is a toxoid preparation, not a live virus. Administration to women in their childbearing years maintains the protection afforded by childhood immunization. Tetanus is an uncommon disease in

*Correspondence to: Dr. William J. Ledger, Department of Obstetrics and Gynecology, New York Hospital-Cornell Medical Center, 525 East 68th Street, J-130, New York, NY 10021.

Received 1 October 1997

Accepted 21 October 1997

TABLE 1. Storch[5]

| | |
|---|---|
| S | Syphilis |
| T | Toxoplasmosis |
| O | Other |
| | Bacterial vaginosis |
| | *Trichomonas vaginalis* |
| | Group B streptococcus |
| | *Escherichia coli* |
| | *Ureaplasma urealyticum* |
| | *Hemophilus influenzae* |
| | Varicella |
| | *Listeria monocytogenes* |
| R | Rubella |
| C | Cytomegalovirus |
| H[5] | Herpes |
| | Human immunodeficiency virus |
| | Hepatitis B |
| | Human papilloma virus |
| | Human parvovirus |

the United States, but it still occurs, particularly among the elderly, and it is preventable.[1] Diphtheria has become a major health problem in Eastern Europe, particularly in some of the new countries that have split off from the old United Soviet Socialist Republic. Pregnant women traveling to that area or who have exposure to Eastern European immigrants are at risk for this disease (Weissenbacher, personal communication).

### Varicella

Varicella is a serious disease for a pregnant woman. It can result in a pneumonia that is fatal[2] and transplacental transmission can result in serious fetal problems.[3] A live virus varicella vaccine has been approved in the United States. Pregnant women should be tested for their susceptibility to this infection. Susceptible women can be immunized postpartum with two injections, 1 month apart.

### Rubella

Rubella represents a success story for preventive care by obstetricians. The introduction of this live virus vaccine in the late 1960s resulted in a dramatic decline in the number of rubella and congenital rubella cases in the United States.[4] The current standard of obstetrical practice involves screening for rubella antibody and immunizing susceptible women postpartum. These patients should be warned that adult women can develop a transient arthritis after receiving this live virus immunization.[5]

### Hepatitis B

The obstetrical record of prevention against hepatitis B is lacking. Currently, obstetricians screen pregnant women for the presence of hepatitis B antigen and prepare the pediatrician for treatment of the newborn of the antigen-positive mother with immunoglobulins and hepatitis B vaccine.[6] This strategy to detect a silent infection is commendable, but it is an incomplete approach to the problem. Hepatitis B is a serious infection, which can be sexually transmitted, and it is preventable. The vaccine contains surface antigens, not live virus, and can be administered to mothers at high risk for hepatitis B acquisition during pregnancy.[7] We believe all susceptible pregnant women should be immunized postpartum. This requires antibody screening for susceptibility and a three-injection immunization schedule given over 6 months in the postpartum period.

### Pneumococcal Pneumonia

Pneumococcal pneumonia can be a serious life-threatening disease. This is particularly true for high risk populations, which include patients over the age of 65 years, and those women with immune deficiencies, with sickle cell disease, or a prior history of a splenectomy. In the past, there was a total physician reliance upon antibiotics, because *Streptococcus pneumoniae* was so susceptible to antibiotics. The recent emergence of ever increasing numbers of penicillin resistant *S. pneumoniae* strains[8] puts this strategy in question. For pregnant women, pneumococcal vaccine should be offered to human immunodeficiency virus (HIV)-positive patients, those with sickle cell disease, and those with a previous history of a splenectomy.

### PREVENTION

Prevention is an unfamiliar therapeutic strategy for obstetricians. It requires knowledge of the natural course of infection, antibody testing for susceptibility, and instruction to avoid disease acquisition during pregnancy.

### Toxoplasmosis

For some reason, screening for toxoplasmosis is not recommended for American obstetricians.[9] This is in contrast to Belgium[10] and France,[11] where screening for toxoplasmosis is not only accepted, but mandated. Arguments in the United States

**TABLE 2. Recommendations for the toxoplasmosis antibody-negative pregnant patient**

- Patients should be advised not to eat raw or undercooked meat, particularly pork, lamb, or venison. Specifically, meat should be cooked to an internal temperature of 150°F (65.5°C); meat cooked until no longer pink inside generally has an internal temperature of 165°F (73.8°C).
- Patients should be advised to wash their hands after contact with raw meat and after gardening or contact with soil; in addition, they should wash fruit and vegetables well before eating them raw.
- If the patient owns a cat, someone else should change the litter box daily; alternatively, the patient should wash her hands thoroughly after changing the litter box. Patients should be encouraged to keep their cats inside and not to adopt or handle stray cats.

**TABLE 3. Recommendations for the CMV antibody-negative pregnant patient**

- Patients should be advised that CMV is shed in semen, cervical secretions, and saliva. Latex condoms should be used during sexual contact, if the patient is not in a monogamous relationship.
- Providers of child care or parents of children in the child care center should be informed that they are at increased risk of acquiring CMV infection. The risk of CMV infection can be diminished by avoiding mouth-to-mouth kissing. Frequent hand washing should be done, particularly after changing diapers.
- If a blood transfusion is needed, these patients should receive only CMV antibody-negative blood or leukocyte-reduced cellular blood products in non-emergency situations.

against toxoplasmosis screening are based upon cost effectiveness. The patient at risk is the susceptible woman who acquires the infection during pregnancy. Although acquisition of toxoplasmosis during pregnancy is uncommon, approximately 90% of those who get infected during pregnancy have no symptoms and thus are unaware of the infection.[12] This makes a strong argument for universal screening of pregnant patients. Suggestions that most mothers who become infected will seek medical care[9] fly in the face of reported reality.[12] This is an uncommon infection. One study of pregnant women in New York City reported 6 toxoplasmosis infections among 4,048 pregnant women (1.5/1,000).[13] Not every newborn exposed to this pathogen will have persistent infection at birth. In a study in Massachusetts, only 52 of 635,000 infants delivered had congenital infection.[14] That is less than 1 in 10,000 births. However, this is a serious infection for the newborn, it is treatable, and only 2 of the 52 infected infants were detected on clinical grounds. Without universal screening, 50 would have been denied early treatment. Despite the infrequency, we believe toxoplasmosis screening is indicated. For susceptible women, a series of preventive guidelines can be given (see Table 2).[15] A similar preventive program in Belgium has significantly reduced maternal acquisition of toxoplasmosis during pregnancy.[10]

### Cytomegalovirus (CMV)

CMV presents a different set of circumstances. It is a much more common infection and most authorities think it is the most frequent pathogen involved in newborn morbidity. A recent publication from the Centers for Disease Control (CDC) estimated that 30,000–40,000 infants are infected each year with CMV and 9,000 of these infants suffer permanent sequelae.[16] Other evaluations indicate that the most frequent and serious newborn infections occur in susceptible women who acquire their first infection during pregnancy.[17] The presence of antibody offers protection to the newborn. American obstetricians have not offered screening for CMV because no effective antiviral therapy has surfaced. This is a short-sighted approach. Antibody screening should be done in pregnant women to determine susceptibility. If susceptible, the pregnant women should have three options presented to her (see Table 3).[15] For this virus, there are a wide variety of risks. CMV is a sexually transmitted disease. A sexually active pregnant woman who is not in a monogamous relationship should require her male sexual partner to wear a condom. This requires attitudinal changes by both the obstetrician and the pregnant woman. The condom in this situation is not to prevent pregnancy, but to lower the risk of acquiring a sexually transmitted disease. Small children in a group setting such as a day care center are frequently infected with CMV and are asymptomatic.[18] They shed large amounts of virus their saliva and urine. Susceptible women should avoid "sloppy" kisses with these children and should wash their hands if they have to change a diaper. Finally, for the rare occasions when a susceptible pregnant woman needs a blood transfusion, the donated blood should be CMV antibody negative. If this is not possible, leukocyte depleted blood should be given to lower the risk of CMV acquisition.[19] None of these preventive measures should be difficult to institute. The starting point in this preventive strategy is antibody screening to determine susceptibility.

### HIV

HIV is an increasingly important problem worldwide for obstetricians because of its frequency and serious long-term implications. In the United States, the number of women infected by heterosexual contact continues to increase and the result is a continuing increase in the number of women developing acquired immunodeficiency syndrome (AIDS).[20] Screening to determine susceptibility is important. If these susceptible women are intravenous drug users, they must be counseled to avoid needle sharing. If they are not in a monogamous sexual relationship or the HIV antibody status of their partner is not known, they should be counseled to require condom use. Again, this is for disease prevention, not contraception.

### Herpes

This is a developing new story for obstetricians. In the past decade, a number of studies have documented the fact that herpes simplex virus (HSV) acquisition can occur without symptomatology.[21,22] These people can shed virus without symptoms and infect susceptible sexual partners. For the pregnant woman, the greatest risk of newborn infection with herpes occurs when the infection is first acquired shortly before the onset of labor. A recent study indicates that this acquisition can be best documented by repeated HSV antibody screening during pregnancy.[23] In this study, only 36% of the women who seroconverted had symptoms consistent with herpes infection. This is a higher incidence of symptoms with virus acquisition than is seen in the non-pregnant adult population.[21] There are interesting future preventive strategies for susceptible women. The male of the herpes susceptible pregnant patient can be tested. If the male is HSV-1 or HSV-2 antibody positive, the patient is at risk for herpes acquisition during pregnancy. Abstinence or the use of a condom during the last trimester may lower the risk for such a patient.[23] Future studies will determine the benefits of such a strategy.

### *Listeria monocytogenes*

*L. monocytogenes* is an uncommon cause of newborn infection, but maternal infection can result in preterm labor and delivery with newborn sepsis and death.[24] Most outbreaks are associated with maternal ingestion of contaminated dairy products. To avoid this, we advise pregnant women in New York City to avoid the ingestion of all cheeses that have not been labeled pasteurized. This includes imported cheeses with a rind as well as feta cheese.

### SILENT INFECTION

Screening and treating silent infections are relatively new concepts for the obstetrician. In the past, we have focused our efforts upon the recognition of the signs and symptoms of a clinical infection, confirming our clinical suspicions with specific laboratory testing. It is becoming increasingly clear that many maternal infections that can cause problems for the mother or fetus are either asymptomatic or cause such minimal symptoms that the mother to be will not seek medical care. This requires physicians to shift their concerns to screen asymptomatic women to detect these infections followed by a treatment strategy. There are a number of screens that should be regularly employed.

### Asymptomatic Bacteriuria

This is a familiar screen for asymptomatic disease by the obstetrician. A clean voided urine is obtained at the first prenatal visit and those women with a colony count of 100,000 or greater are treated. There is good evidence that this strategy reduces the subsequent incidence of pyelonephritis.[25] This has been an effective preventive strategy for obstetricians.

### Bacterial Vaginosis (BV)

This is the current infectious disease "hot item" for obstetricians. BV is manifested by a tremendous increase in the number of bacteria in the vagina and many women so detected are asymptomatic. More important, the Vaginal Infections in Pregnancy (VIP) study showed an association with BV and the delivery of a low birth weight infant.[26] An optimistic sign is that treatment studies with both systemic clindamycin[27] and metronidazole[28] have been effective in reducing the rates of preterm births. Vaginal antibiotic preparations which are effective in the treatment of BV have not resulted in a lowering of the rate of prematurity.[29,30] Armed with this information, a system of screening and treatment of those who test positive has been devised.

The diagnosis of BV can be made within minutes of the obstetrician-patient contact. Every time a vaginal examination is done on a pregnant woman, the following screening tests should be done. The vaginal pH should be tested to see if it is alkaline and a drop of vaginal secretion placed on a drop of dilute potassium hydroxide on a slide and checked immediately to see if a "fishy" odor emanates. If either of these tests is positive, another vaginal secretion sample should be placed on a drop of normal saline on a slide and examined under a microscope. If over 20% of the epithelial cells are "clue" cells, the diagnosis is confirmed. The treatment should be with oral metronidazole[28] or oral clindamycin[27] for at least 7 days. There is one study from Germany suggesting that vaginal clindamycin treatment given before 14 weeks gestation lowers the incidence of preterm births in the treated population (Dennemark, personal communication). Further studies on this early pregnancy population will be needed to confirm this observation. If so, it would simplify the care of women with BV in the first trimester.

### *Trichomonas vaginalis*

There is increasing interest in the asymptomatic pregnant patient with *T. vaginalis* vaginitis. In the VIP study, the finding of *T. vaginalis* by a culture technique was associated with a higher incidence of preterm labor than was seen with BV.[31] Another recent study using the polymerase chain reaction (PCR), a more sensitive test than the culture technique used in the VIP study, found a high incidence of vaginal *Trichomonas* infection in an urban poor population with a high incidence of prematurity.[32] Further information about the risk of *Trichomonas* for the pregnant patient requires a dual strategy of physician education and prospective study. Physicians must learn that asymptomatic vaginal *Trichomonas* exists and the microscopic examination of vaginal secretions in saline is not a sensitive screening test.[33] Universal screening needs to be done on pregnant populations, comparing the pregnancy outcomes in PCR and culture-positive patients. Finally, this infected population needs to be treated with metronidazole to determine if the preterm delivery rate is decreased.

### *Neisseria gonorrhoege*

Pregnant patients can be culture positive for *N. gonorrhoeae* without enough symptomatology to cause them to seek medical attention. If untreated, these patients have a higher incidence of preterm labor and delivery.[34] Part of pregnancy care should involve screening for *N. gonorrhoeae.* This can be done with a DNA probe for the incidence if penicillin resistance is high enough that cephalosporins are now recommended. Treatment with a single dose of ceftriaxone 250 mg 1M should be adequate.[35] Antibiotic treatment of the pregnant woman is also important, because an infected mother can infect her newborn's eyes through the process of labor and delivery.

### *Chlamydia trachomatis*

*C. trachomatis* is a good example of a bacterial pathogen that can cause serious problems for both the mother and the fetus. Women infected with *C. trachomatis* have no symptoms or minimal symptomatology that is not severe enough for them to seek medical care. These symptom-free women, if infected, have the potential for a series of problems for the pregnant patient and the fetus. Pregnant women with *C. trachomatis* antibodies, evidence of a past infection, have a higher than expected incidence of spontaneous abortion.[36] Among premature newborns delivered of women colonized with *C. trachomatis,* there is a risk of developing a *C. trachomatis* pneumonia.[37] The newborns of culture-positive women, both premature and at term, have the potential for developing ophthalmia neonatorum. These serious problems and lack of patient symptomatology demand universal screening of pregnant women. PCR should be used because it is so much more sensitive and specific than the DNA probes.[38] PCR-positive women and their sexual partners should be treated. The CDC recommends 7 days of oral erythromycin therapy for the pregnant woman, for it is safe during pregnancy.[35] The problem is that erythromycin is poorly tolerated by most pregnant women. They have enough gastrointestinal distress that many of these asymptomatic women do not complete their course of treatment. For this reason, we favor the use of amoxicillin in those pregnant women who are not allergic to penicillin. This antibiotic is better tolerated by the pregnant recipient and it is effective in eliminating the organism.[39] In addition to screening at the time of the first prenatal visit, we favor another PCR screen at 35–37 weeks gestation. Term infants born of infected mothers can develop ophthalmia neonatorum from the *C. trachomatis* and the antibiotic ophthalmic ointments used in the newborn are not very effective in curing the infection.[40] These culture-positive women should be treated prior to delivery.

### Group B Streptococcus

The group B streptococcus is a feared organism by obstetricians. Vaginal colonization with this organism which causes no maternal symptomatology can result in vertical transmission to the fetus with newborn sepsis and death despite newborn antibiotic treatment.[41] There is evidence that treatment of the mother with antibiotics prior to delivery markedly reduces newborn infectious morbidity and mortality.[42] The problem with any strategy of antibiotic prophylaxis is the selection of the mother at risk for newborn group B streptococcal infection.

Much is known about maternal colonization and newborn infections with the group B streptococcus. Vaginal colonization with the group B streptococcus is not a constant. Women colonized in the first, second, or third trimester may not be culture positive at the time of delivery. Alternatively, women culture negative at the first, second, or third trimester vaginal culture screening may be culture positive at the time of delivery. In one study, only 51% of women culture positive in the first trimester remained culture positive at the time of delivery.[43] This raises natural concerns about preventive strategies using antepartum cultures. Some women who are culture positive with an antepartum screen will be negative at the time of birth and some women who are culture positive at the time of delivery will not be detected at the time of the antepartum screen. Rapid screening for the group B streptococcus would obviate this problem, but the systems studied to date have not been sensitive or specific.[44] This is not an acceptable option at present. Another problem is the discrepancy between the incidence of maternal colonization and newborn infection. This varies between 1:20[45] and 1:102,[46] with the highest incidence of newborn problems seen in those women who deliver premature infants. This means many mothers will be treated to prevent one case of newborn sepsis. No one recommends treating antepartum culture-positive patients with antibiotics, even though there is an association with first trimester group B streptococcus colonization and preterm premature rupture of the membranes and preterm delivery.[47] There are two major reasons for this. This antibiotic intervention has not proved successful in lowering the premature delivery rate[48] and systemic antibiotic treatment has not been effective in the persistent eradication of the group B streptococcus from the vagina of these women.[49] Finally, there is the question of the selection of pregnant women to receive antibiotic prophylaxis in labor. The CDC has suggested two strategies for the patient who is not penicillin allergic; they recommend penicillin G with ampicillin as an alternative.[50] There is controversy surrounding both of these choices. Penicillin G has a narrow range of antibacterial activity. For the obstetrician concerned about other organisms which cause newborn infection with some frequency such as *Escherichia coli, Hemophilus influenzae, Listeria monocytogenes, Ureaplasma urealyticum*, and *C. trachomatis* are not covered. On the positive side, widespread use of this narrow spectrum antibiotic should not have a major impact on the bacterial flora found on a labor and delivery floor. Many obstetricians favor ampicillin, because its wider spectrum of activity means that some strains of *E. coli, H. influenzae*, and *L. monocytogenes* will be covered as well as the group B streptococcus. This widespread use of a broader spectrum antibiotic has a cost. There will be a greater impact upon the antibiotic flora of an obstetrical service and there already has been one report of an increase in the number of gram-negative aerobic infections in the newborn when this ampicillin prophylaxis was employed.[51] There are important considerations to be kept in mind when a preventive regimen is proposed. All antibiotic intervention regimens have the capability of lowering the incidence of group B streptococcal disease.[52] All are different in their technique of selecting mothers at risk (see Table 4) and none is 100% successful in preventing group B streptococcal newborn disease.[52] Intrapartum antibiotic administration to the mother will not prevent all cases. There have been instances in which mothers given antibiotics when they were admitted in labor still have delivered infants with group B sepsis and death due to this infection.[50,52]

All of this is prologue to the current CDC rec-

TABLE 4. Strategies for the prevention of early onset neonatal group B streptococcal infection

| Strategy | Screening | Treatment |
|---|---|---|
| 1 | None | None |
| 2 | None | All |
| 3 | None | All high risk (HR) |
| 4 | Culture at 28 weeks | All culture positive (CP) |
| 5 | Culture at 28 weeks | All (CP) (HR) |
| 6 | Culture at 28 weeks | All (CP) (HR) |
| 7 | Culture at 36 weeks | All preterm, all (CP) |
| 8 | Culture at 36 weeks | All preterm, all (CP) (HR) |
| 9 | All rapid tests (RT) in labor | All (RT) positive |
| 10 | (RT) (HR) in labor | All (RT) positive |
| 11 | (RT) low risk (LR) in labor | All (HR), all (RT) positive |
| 12 | Culture at 28 weeks; (RT) culture negative (HR) | All (HR), (RT) positive |
| 13 | Culture at 28 weeks; (RT) all culture negative | All (CP), all (RT) positive |
| 14 | Culture at 28 weeks; (RT) (LR) (CP) | All (HR), all (RT) positive |
| 15 | Culture at 28 weeks; (RT) culture negative (LR) | All (HR), all low risk (CP), (RT) positive |
| 16 | Culture at 28 weeks; (RT) (LR) (CR) | (CP) (HR), (RT) positive |
| 17 | Culture at 28 weeks; (RT) (LR) (CP); (RT) (HR) culture negative | CP (HR), (RT) positive |
| 18 | Culture at 28 weeks; (RT) all (LR); (RT) culture negative (HR) | CP (HR), (RT) positive |
| 19 | Culture at 28 weeks; (RT) culture negative (HR) | All CP, all (RT) positive |

ommendations for the control of the group B streptococcus (Figs. 1, 2).[50] The recommendations fit into two main strategies. Each identifies group B streptococcal risk factors. These include the woman who has had a previous infant with group B streptococcal disease, group B streptococcal bacteriuria during this pregnancy, and delivery at less than 37 weeks gestation. The divergence involves antepartum screening for vaginal carriage of the group B streptococcus to identify women who should receive intrapartum antibiotics. This requires medical team sampling of the lower third of the vagina and the rectum with the same swab and inserting this specimen in a selective culture medium to increase the yield of positive group B streptococcus cultures. Whether this increased group B streptococcal laboratory recovery equates with improved newborn results is not known. Alternatively, no antepartum cultures are done. Risk factors identify mothers who will be given intrapartum antibiotics. These include the three previously mentioned: antepartum asymptomatic bacteriuria with the group B streptococcus, women with a history of a previous baby who developed group B streptococcus sepsis, and those with preterm premature rupture of the membranes and preterm labor and delivery. There are additional high risk factors for women beyond 37 weeks. They should be treated intrapartum with penicillin if they develop a fever during labor, i.e., $\geq$38°C orally, or those who will have membranes ruptured $\geq$18 h at the time of delivery. There have been questions raised about the $\leq$37 week definition of prematurity, the 38°C oral temperature cutoff, and 18 h definition of prolonged membrane rupture. There may be another risk factor for group B streptococcal newborn infections. Two studies have shown a correlation with the use of a fetal scalp electrode during labor and the subsequent development of a newborn group B streptococcal infection.[53,54] Further observations will be needed to fully determine the risk of this invasive monitoring for newborn group B streptococcal infection. With all of these concerns, Figures 1 and 2 represent the current standards for prevention for the obstetrician in the United States.

We have two major reservations about the CDC recommendations. They enforce a practice pattern that may not pass the test of time. I have no doubt these recommendations will increase the use of intrapartum antibiotics in the United States and decrease the number of group B streptococcal newborn infections in the short run. Over the long term, this strategy increases the risk of altering bacterial flora causing newborn disease. Surveillance of the organisms causing newborn sepsis will be necessary to detect this. These guidelines will also limit the effectiveness of prospective studies trying to pinpoint mothers at risk for having babies with newborn disease and it will impede the study of alternative approaches. For example, some obstetrical services in Europe use an antiseptic vaginal

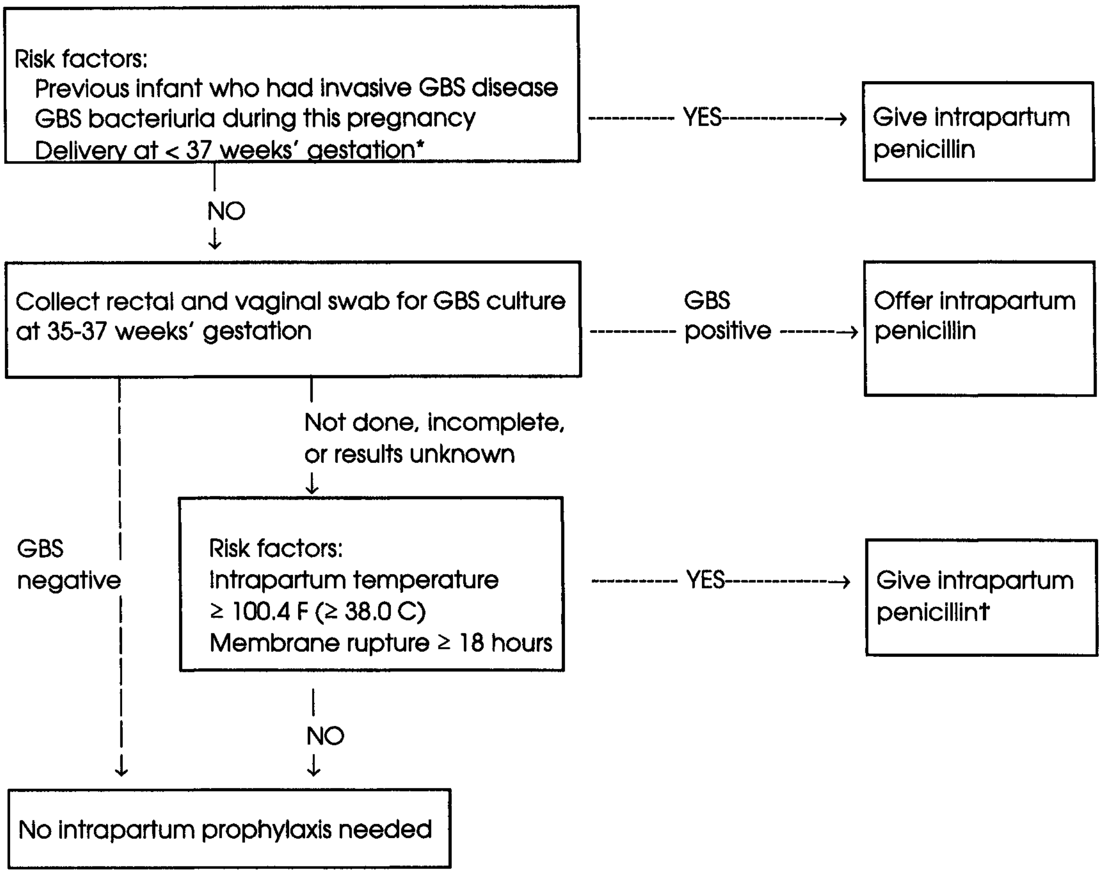

* If membrane ruptured at < 37 weeks' gestation, and the mother has not begun labor, collect group B streptococcal culture and either a) administer antibiotics until cultures are completed and the results are negative or b) begin antibiotics only when positive cultures are available. No prophylaxis is needed if culture obtained at 35-37 weeks' gestation was negative.

† Broader spectrum antibiotics may be considered at the physician's discretion, based on clinical indications.

Fig. 1. Algorithm for prevention of early onset of group B streptococcal (GBS) disease in neonates, using prenatal screening at 35–37 weeks gestation.

lavage on all women admitted in labor and report a remarkable decrease in group B streptococcal newborn disease (Stray-Pederson, personal communication).

### *E. coli*

*E. coli* is another pathogen of concern for obstetricians in their quest to control newborn infections. It is a frequent cause of newborn infection[55] and on most services ranks next to the group B streptococcus in frequency. There are some aspects of *E. coli* newborn infection that lend themselves to strategies of control. Newborn infection results from vertical transmission from the mother. Unlike the group B streptococcus, maternal vaginal colonization with *E. coli* seems to be a constant.[56] This suggests that antepartum vaginal screening for this organism could identify those women at risk. In addition, specific strains of *E. coli* seem to have more invasive properties and to be more dangerous for newborns.[57] Again, identification screening techniques could be employed to delineate women at risk for infection. Intrapartum treatment should be effective, but many *E. coli* strains are resistant to

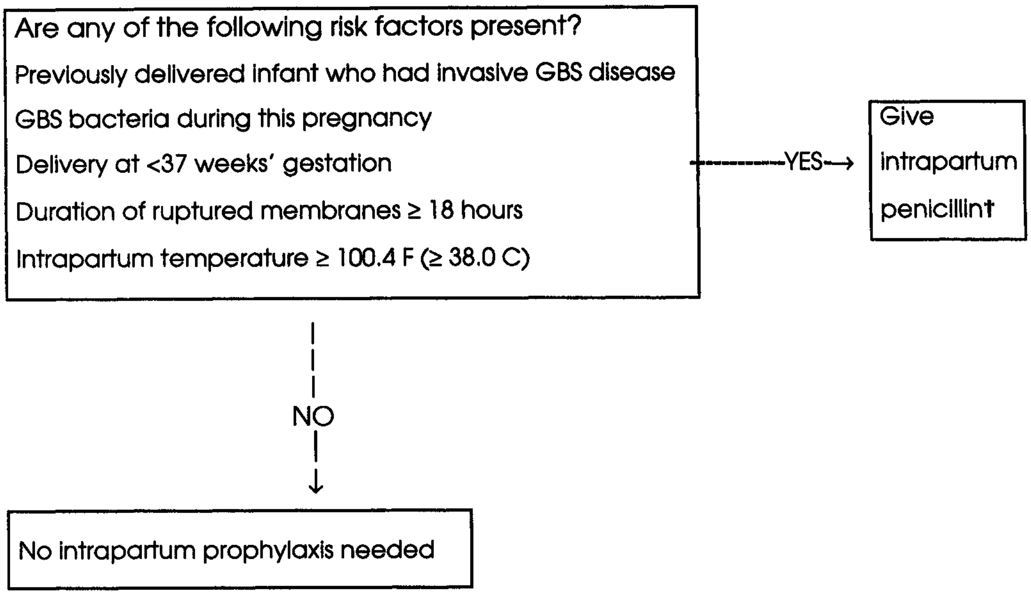

* If membrane ruptured at <37 weeks' gestation, and the mother has not begun labor, collect group B streptococcal culture and either a) administer antibiotics until cultures are completed and the results are negative or b) begin antibiotics only when positive cultures are available.

† Broader spectrum antibiotics may be considered at the physician's discretion, based on clinical indications.

Fig. 2. Algorithm for prevention of early onset of group B streptococcal (GBS) disease in neonates, using risk factors.

ampicillin. Prospective studies need to be done to determine the effectiveness of cephalosporins or ampicillin with a beta lactamase inhibitor added.

### H. influenzae

*H. influenzae* is another important cause of newborn infection.[58] A current therapeutic problem is that many strains of *H. influenzae* are beta lactamase producers and as such are resistant to ampicillin. Alternative non-beta lactam antibiotics would be better first-line agents in hospitals where ampicillin resistant *H. influenzae* is a frequent finding.

### U. urealyticum

*U. urealyticum* remains an enigma. It is associated with chronic lung disease in the premature newborn.[59] The difficulty is that the majority of pregnant women are colonized with this organism[60] so the problem of determining which newborn of these colonized women will be at risk remains unsolved. This organism has the potential to cause problems. It can occasionally be isolated from the amniotic fluid of women in premature labor.[61] In evaluating the pregnancy history of newborns who become infected, the route of delivery did not alter the risk of infection, but chorioamnionitis did.[62] The antibiotic of choice in the past has been erythromycin, but administration of this drug to pregnant women was ineffective in reducing the number of women who remained vaginal culture positive for this organism.[60] Clearly, more studies will be needed to delineate risk factors.

### REFERENCES

1. CDC: Tetanus surveillance—United States, 1991–1994, MMWR 46:15–25, 1997.
2. Harris RD, Rhoades EF: Varicella pneumonia complicating pregnancy. Report of a case and review of the literature. Obstet Gynecol 25:734–740, 1965.
3. Pastuszak AL, Levy M, Schick B, et al.: Outcome of the maternal varicella infection in the first 20 weeks of pregnancy. N Engl J Med 330:901–905, 1994.
4. CDC: Increase in rubella and congenital rubella syndrome—United States, 1988–1990. 40:93–99, 1991.
5. Howson CP, Katz M, Johnson RB Jr, et al.: Chronic arthritis after rubella vaccination. Clin Infect Dis 15: 307–312, 1992.
6. Stevens CE, Taylor PE, Tong MJ, et al.: Yeastrecombinant-hepatitis B vaccine. JAMA 257:2612–2618, 1987.

7. Faro S: To vaccinate or not to vaccinate. Infect Dis Obstet Gynecol 2:153, 1994.
8. Butler JC, Hofmann J, Catron MS, et al.: The continued emergence of drug-resistant *Streptococcus pneumoniae* in the United States, an update from the Centers for Disease Control and Prevention's pneumonococcal sentinel surveillance system. J Infect Dis 174:986–993, 1996.
9. American College of Obstetricians and Gynecologists: Perinatal viral and parasitic infection. Tech Bull 177:1–7, 1993.
10. Foulon W, Naessens A, Derde MD: Evaluation of the possibilities for preventing congenital toxoplasmosis. Am J Perinatol 11:57–62, 1994.
11. McCabe R, Remington JS: Toxoplasmosis: The time has come. N Engl J Med 318:313–315, 1988.
12. Wilson CB, Remington JS: What can be done to prevent congenital toxoplasmosis? Am J Obstet Gynecol 138: 357–363, 1980.
13. Kimball AC, Kean BH, Fuchs F: Congenital toxoplasmosis: A prospective study of 4,048 obstetric patients. Am J Obstet Gynecol 111:211–218, 1971.
14. Guerina NG, Hsu H-W, Meissner C: Neonatal serologic screening and early treatment for congenital *Toxoplasma gondil* infection. N Engl J Med 330:1858–1863, 1994.
15. CDC: US PHS/IDSA guidelines for the prevention of opportunistic infections in persons infected with human immunodeficiency virus: A summary. MMWR 44:1–34, 1995.
16. Demmler GJ: Summary of a workshop on surveillance for congenital cytomegalovirus disease. Rev Infect Dis 13:315–329, 1991.
17. Stagno S, Pass RE, Henderson RE, et al.: Congenital cytomegalovirus infection. The relative importance of primary and recurrent maternal infection. N Engl J Med 306:945–949, 1982.
18. Pass RE, Hutto C, Ricks R, et al.: Increased rate of cytomegalovirus infection among parents of children attending day care centers. N Engl J Med 314:1414–1418, 1986.
19. Gilbert GL, Hayes K, Hudson IL, et al.: Prevention of transfusion-acquired cytomegalovirus infection in infants by blood filtration to remove leukocytes. Lancet 1:1228–1231, 1989.
20. CDC: Update: Trends in AIDS incidence, deaths, and prevalence—United States, 1996. MMWR 46:168–173, 1997.
21. Breining MK, Kingsley LA, Armstrong JA, et al.: Epidemiology of genital herpes in Pittsburgh: Serologic, sexual and racial correlates of apparent and inapparent herpes simplex infections. J Infect Dis 162:299–305, 1990.
22. Kulhanjian JA, Soroush V, Au DS, et al.: Identification of women at unsuspected risk of primary infection with herpes simplex virus type 2 during pregnancy. N Engl J Med 326:916–920, 1992.
23. Brown ZA, Selke S, Zeh J, et al.: The acquisition of herpes simplex virus during pregnancy. N Engl J Med 337:509–515, 1997.
24. Linnan MJ, Mascola L, Lou KD, et al.: Epidemic listeriosis associated with Mexican style cheese. N Engl J Med 319:823–828, 1988.
25. Harris RE: The significance of eradication of bacteriuria during pregnancy. Obstet Gynecol 53:71–73, 1979.
26. Hillier SL, Nugent RP, Eschenbach DA, et al.: Association between bacterial vaginosis and pre-term delivery of a low-birth-weight infant. N Engl J Med 333: 1737–1742, 1995.
27. McGregor JA, French JI, See K: Adjunctive clindamycin therapy for preterm labor: Results of a double-blind, placebo controlled trial. Am J Obstet Gynecol 165:867–875, 1991.
28. Morales WJ, Schorr S, Albritton J: Effect of metronidazole in patients with preterm birth in preceding pregnancy and bacterial vaginosis: A placebo-controlled, double blind study. Am J Obstet Gynecol 171:345–349, 1994.
29. McGregor JA, French JI, Jones W, et al.: Bacterial vaginosis is associated with prematurity and vaginal fluid mucinase and sialidase: Results of a controlled trial of topical clindamycin cream. Am J Obstet Gynecol 170: 1048–1060, 1994.
30. Joesoef MR, Hillier SL, Wiknjostro G, et al.: Intravaginal clindamycin treatment for bacterial vaginosis: Effects on preterm delivery and low birth weight. Am J Obstet Gynecol 173:1527–1531, 1995.
31. Pastorek JG, Cotch MF, Martin DH, et al.: Clinical and microbiological correlates of vaginal *Trichomonas* during pregnancy. Clin Infect Dis 23:1075–1080, 1996.
32. Jeremias J, Draper D, Ziegert M, et al.: Detection of *Trichomonas vaginalis* using the polymerase chain reaction in pregnant and non-pregnant women. Infect Dis Obstet Gynecol 2:16–19, 1994.
33. Fouts AC, Kraus SJ: *Trichomonas vaginalis:* Reevaluation of its clinical presentation and laboratory diagnosis. J Infect Dis 141:137–143, 1980.
34. Elliot B, Brunham RC, Laga M, et al.: Maternal gonococcal infection as a preventable risk factor for low birth weight. J Infect Dis 161:531–536, 1990.
35. CDC: 1993 sexually transmitted diseases treatment guidelines. MMWR 42:1–102, 1993.
36. Licciardi F, Grifo JA, Rosenwaks Z, et al.: Relation between antibodies to *Chlamydia trachomatis* and spontaneous abortion following in vitro fertilization. J Assist Reprod Genet 9:207–210, 1992.
37. Alexander ER, Harrison HR: Role of *Chlamydia trachomatis* in perinatal infection. Rev Infect Dis 5:713–719, 1983.
38. Witkin SS, Jeremias J, Toth, et al.: Detection of *Chlamydia trachomatis* by the polymerase chain reaction in the cervices of women with acute salpingitis. Am J Obstet Gynecol 168:1438–1442, 1993.
39. Crombleholme WR, Schachter J, Grossman M, et al.: Amoxicillin therapy for *Chlamydia trachomatis* in pregnancy. Obstet Gynecol 75:752–756, 1990.
40. Hammerschlag MR, Cummings C, Roblin PM, et al.:

Efficacy of neonatal ocular prophylaxis for the prevention of chlamydia and gonococcal conjunctivitis. N Engl J Med 320:769–772, 1989.

41. Pyati SP, Plides RS, Jacobs NM, et al.: Penicillin in infants weighing two kilograms or less with early onset group B streptococcal disease. N Engl J Med 308:1383–1389, 1983.
42. Yow MD, Mason EO, Leeds LJ, et al.: Ampicillin prevents intrapartum transmission of group B streptococcus. JAMA 241:1245–1247, 1979.
43. Lewin EB, Amstey MS: Natural history of group B streptococcus colonization and its therapy during pregnancy. Am J Obstet Gynecol 139:512–515, 1981.
44. Skoll MA, Mercer BM, Baselski V, et al.: Evaluation of two rapid group B streptococcal antigen tests in labor and delivery patients. Obstet Gynecol 77:322–326, 1991.
45. Franciosi RA, Knostman JD, Zimmerman RA: Group B streptococcal neonatal and infant infections. J Pediatr 82:707, 1973.
46. Baker CJ: Group B streptococcal infections in neonates. Pediatr Rev 64:5, 1979.
47. Regan JA, Chao S, James LS: Premature rupture of membranes, preterm delivery and group B streptococcal colonization of mothers. Am J Obstet Gynecol 141:184–186, 1981.
48. Klebanoff MA, Regan JA, Rao AV, et al.: Outcome of the vaginal infections and prematurity study: Results of a clinical trial of erythromycin among pregnant women colonized with group B streptococci. Am J Obstet Gynecol 172:1540–1545, 1995.
49. Hall RL, Barnes W, Krishnan L, et al.: Antibiotic treatment of parturient women colonized with group B streptococci. Am J Obstet Gynecol 124:630–634, 1976.
50. CDC: Prevention of perinatal group B streptococcal disease: A public health perspective. MMWR 45:1–24, 1996.
51. McDuffie RS, McGregor JA, Gibbs RS: Adverse prenatal outcome and resistant Entro bactriacase after antibiotic usage for premature rupture of membranes and group B streptococcus carriage. Obstet Gynecol 22:487–489, 1993.
52. Rouse DJ, Goldenberg RL, Cliver SP, et al.: Strategies for the prevention of early onset neonatal group B streptococcal sepsis: A decision analysis. Obstet Gynecol 83: 483–494, 1994.
53. Bobitt JR, Ledger WJ: Obstetric observations in eleven cases of neonatal sepsis due to the group B beta hemolytic streptococcus. Obstet Gynecol 47:439–442, 1976.
54. Gill P, Sobeck J, Jarjoura D, et al.: Mortality from early onset neonatal group B streptococcal sepsis: Influence of obstetric factors. J Mat Fet Med 6:35–39, 1997.
55. Gladstone IM, Ehren Kranz RA, Edberg SC, et al.: A ten year review of neonatal sepsis and comparison with previous fifty year experience. Pediatr Infect Dis J 9: 819–825, 1990.
56. Amstey MS, Lewin E, Colaice J: Vaginal colonization with invasive *Escherichia coli* during pregnancy. Am J Obstet Gynecol 137:534–535, 1980.
57. Robbins JB, McCracken GH, Gotschlick EC, et al.: *Escherichia coli K.* capsular polysaccharide associated with neonatal meningitis. N Engl J Med 290:1216, 1974.
58. Rusin P, Adam RD, Petersen EA, et al.: *Hemophilus influenzae:* An important cause of maternal and neonatal infections. Obstet Gynecol 77:92–96, 1991.
59. Payne NR, Steinberg SS, Acherman P, et al.: New prospective studies of the association of *Ureaplasma urealyticum* colonization and chronic lung disease. Clin Infect Dis 17:S117–S121, 1993.
60. Eschenbach DA, Nugent RP, Rao AV, et al.: A randomized placebo controlled trial of erythromycin for the treatment of *Ureaplasma urealyticum* to prevent premature delivery. Am J Obstet Gynecol 164:734–742, 1991.
61. Eschenbach DA: *Ureaplasma urealyticum* and premature birth. Clin Infect Dis 17:S100–S106, 1993.
62. Sanchez PJ: Perinatal transmission of *Ureaplasma urealyticum:* Current concepts based on review of the literature. Clin Infect Dis 17:S107–S111, 1993.
